# A Study of Calcium-Silicate-Hydrate/Polymer Nanocomposites Fabricated Using the Layer-By-Layer Method

**DOI:** 10.3390/ma11040527

**Published:** 2018-03-30

**Authors:** Mahsa Kamali, Ali Ghahremaninezhad

**Affiliations:** Department of Civil, Architectural and Environmental Engineering, University of Miami, Coral Gables, FL 33146, USA; m.kamali@umiami.edu

**Keywords:** CSH, LBL, AFM image, polymer

## Abstract

Calcium-silicate-hydrate (CSH)/polymer nanocomposites were synthesized with the layer-by-layer (LBL) method, and their morphology and mechanical properties were investigated using atomic force microscopy (AFM) imaging and AFM nanoindentation. Different sets of polymers were used to produce CSH/polymer nanocomposites. The effect of different factors including dipping time, calcium to silicate ratios (C/S ratios) and pH on morphology was investigated. CSH/polymer nanocomposites made with different sets of polymers showed variation in morphologies. However, the Young’s modulus did not seem to reveal significant differences between the nanocomposites studied here. In nanocomposites containing graphene oxide (GO) nanosheet, an increase in the density of CSH particles was observed on the GO nanosheet compared to areas away from the GO nanosheet, providing evidence for improved nucleation of CSH in the presence of GO nanosheets. An increase in roughness and a reduction in the packing density in nanocomposites containing GO nanosheets was observed.

## 1. Introduction

Improving the mechanical and durability performance of concrete is a way to increase sustainability in infrastructure [1,2]. Calcium-silicate-hydrate (CSH) is the primary phase of hydration product and directly affects the strength and durability properties of cementitious materials [3,4,5,6]. There is an increasing interest in innovative approaches to manipulate the structure of CSH at the nanoscale as a way to influence the macroscale properties [7,8,9,10,11,12,13,14,15]. Use of organic additives to modify the nanostructure of CSH holds potential as a viable approach to influence the properties of CSH. This approach is inspired by the microstructure of certain biological nanocomposites, such as bones, teeth and the nacre of abalone shells, which exhibit superior engineering performance compared to traditional materials [16,17]. Researchers have studied the possibility of modifying the structure of CSH with polymers [7,8,9,11,12,13,14,15,18,19]. Matsuyama et al. [7,8,9] investigated the possibility of intercalation of polymers with different charges into the structure of CSH with various calcium to silicate (C/S) ratios. Pelisser et al. [11,15] showed that poly(diallyldimethylammonium chloride) and poly(vinyl alcohol) can be intercalated between the CSH layers. Khoshnazar et al. [14,20,21] studied the characteristics of CSH modified with nitrobenzoic acid and aminobenzoic acid. Alizadeh et al. [12] achieved CSH/polyaniline nanostructures by polymerizing the aniline monomer in the synthesized CSH/aniline complex. Minet et al. [18] reported that small molecular-sized organic molecules can intercalate between the CSH layers without changing the structural framework of CSH.

The layer-by-layer (LBL) method is a widely-used “bottom-up” nanofabrication technique that has applications in the fabrication of various types of coatings and free-standing thin films and hierarchical nanostructures [22,23,24,25,26,27]. Bottom-up nanofabrication is a novel manufacturing strategy in which the component molecules are self-assembled to build the desired nanostructure [28]. The LBL assembly technique allows different types of polymers and nanoparticles to be incorporated in the structure of thin films through primarily electrostatic interaction [29]. This technique allows fabrication of various composites with a nanoscale control. LBL has numerous applications in the design of light-emitting diodes [30,31], non-linear optics [32], metal composites [33,34], biomedical microcapsules [35,36] and tissue engineering [37,38]. Inorganic materials such as colloidal particles, graphene oxide [39] and nanoclay have been successfully incorporated in nanocomposites fabricated using the LBL technique. Guo et al. [25,26] used CSH as the inorganic unit in LBL assemblies to fabricate superhydrophilic and antireflective coatings. Two polymer/ion complexes, one with calcium ions and one with silicate ions, were deposited layer by layer on a substrate. Precipitation of CSH between the layers was provided through the reaction of calcium and silicate ions. They reported that the fabricated membrane was a superhydrophilic composite that exhibited superior antifogging characteristics.

Due to the ability of the LBL method to produce nanocomposites with a nanoscale control, this technique can be useful in the fundamental study of CSH/polymer nanocomposites. The LBL technique permits the investigation of the effect of various relevant parameters including the polymer molecular structure and charge, CSH composition, pH, and others, on the nanostructure and nanomechanical properties of CSH/polymer nanocomposites. In addition, since the surface characteristics including surface roughness of CSH/polymer nanocomposite can be tuned to achieve a very low roughness suitable for nanoindentation, the LBL technique can provide an alternative approach to fabricating samples for use in the nanomechanical characterization of this kind of nanocomposite.

In spite of the foregoing discussion, studies on the characteristics of CSH/polymer nanocomposites fabricated using the LBL method are very scarce. Thus, this study aims to investigate the morphology and nanomechanical properties of CSH/polymer nanocomposites synthesized using the LBL method. Specifically, CSH/polymer nanocomposites made using different sets of polymers were used to examine the effect of polymers on the characteristics of the nanocomposites.

Graphene oxide (GO) nanosheets with a sheet-like structure and functional groups are considered a promising additive for cementitious materials [1,40]. It is shown that the use of GO nanosheets can improve the mechanical properties of cement paste at the macroscale by modifying the formation and regulating the microstructure of CSH [41,42,43,44]. Therefore, GO nanosheets were, for the first time, incorporated in the LBL-fabricated CSH/polymer nanocomposite to obtain insight into their effect on the nanocomposite. The planar shape of GO is well suited to study its interaction with CSH in LBL-fabricated nanocomposites. 

## 2. Experiments

### 2.1. Materials 

**Figure 1:**
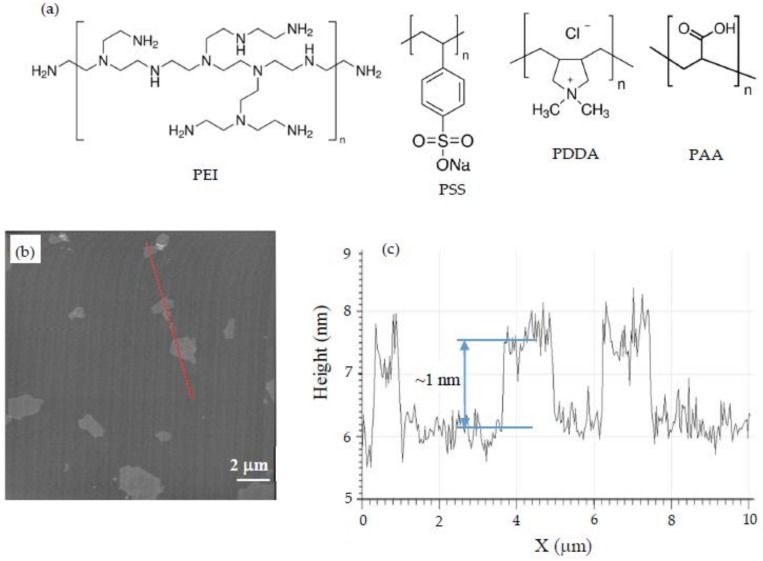
(**a**) Molecular structures of the polymers used; (**b**) AFM scan of the graphene oxide nanosheet on a mica substrate; (**c**) Height profile along the line indicated by a red line in Figure 1b. The thickness of one nanosheet is marked in Figure 1c.

Calcium acetate, sodium silicate and the polymers were purchased from Sigma-Aldrich (St. Louis, MO, USA). Cationic polymers used in this study were poly(ethyleneimine) (PEI) with a molecular weight of M_w_ = 750,000 g/mol and poly(diallyldimethylammonium chloride) (PDDA) with a molecular weight of M_w_ = 200,000–350,000 g/mol. PEI contains amino groups, but PDDA is a quaternary ammonium polymer [45]. The anionic polymers included poly(sodium 4-styrenesulfonate) (PSS) with M_w_ = 70,000 g/mol and poly(acrylic acid) (PAA) with M_w_ = 250,000 g/mol. The molecular structures of the polymers are shown in Figure 1a (adapted from [46]). The graphene oxide (GO) used was purchased in the form of a dispersion with a concentration of 5 mg/mL. To measure the thickness and planar dimensions of the GO nanosheets, a diluted solution of the dispersion was placed on the surface of freshly-cleaved mica and rinsed after a few minutes. The surface of the mica was scanned by AFM. The details of AFM imaging are provided later in the paper. The AFM scan of the mica surface is shown in Figure 1b, and a height profile of a line marked in Figure 1b is shown in Figure 1c. It can be seen that the GO nanosheets are about 1 nm in thickness and 1–2 μm in the planar dimensions.

### 2.2. Sample Preparation

**Figure 2:**
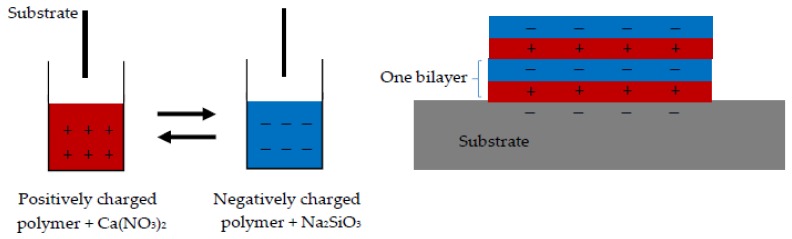
Schematic showing the layer-by-layer (LBL) fabrication of calcium-silicate-hydrate (CSH)/polymer nanocomposites.

An amount of 0.4 g of positively- and negatively-charged polymers was separately dissolved in 200 g of deionized (DI) water. Varied amounts of sodium silicate and calcium acetate were added to the negatively-charged polymer solutions and positively-charged polymer solutions, respectively, to achieve the desired (C/S ratios (mol/mol)) in the LBL-fabricated CSH/polymer nanocomposites. A glass slide was used as the substrate for LBL deposition. Substrates were cleaned with acetone and treated with a hot Piranha solution (3:1 mixture of sulfuric acid and 30% solution of hydrogen peroxide) until they stopped bubbling followed by rinsing with DI water. Piranha solution wipes off any organic residues and provides a negatively-charged surface on the glass slide. First, the substrate was dipped into a solution of positively-charged polymer without calcium acetate for 20 min and then rinsed with DI water. Second, the substrate was dipped into a solution of negatively-charged polymer without sodium silicate for 20 min followed by rinsing with DI water. These two layers served as a cap layer to facilitate the adhesion of the following layers. The LBL deposition was followed by dipping the substrate in the positively-charged polymer solution with calcium acetate, rinsing with DI water, dipping the substrate in the negatively-charged polymer solution with sodium silicate, rinsing with DI water and repeating these steps until the desired number of bilayers were formed on the substrate. A schematic depicting the LBL fabrication is shown in Figure 2. Samples with three bilayers were made for the morphological study. For the nanomechanical testing, samples with 25 bilayers were made to provide sufficient thickness for AFM nanoindentation. For the Fourier transform infrared spectroscopy (FTIR), a polyacrylonitrile (PAN) substrate was used rather than a glass slide in order to allow the detection of the chemical bonds corresponding to CSH. No Piranha solution was used for preparing the PAN substrate. Instead, it was hydrolyzed in a 2 M NaOH solution for an hour and then rinsed thoroughly with deionized water before the LBL fabrication. Samples were also prepared without CSH and labelled PEI/PSS, PDDA/PAA and PEI/PAA. Samples with CSH were labelled PEI/PSS-CSH, PDDA/PAA-CSH and PEI/PAA-CSH. In order to study the effect of dipping time, CSH/polymer nanocomposite samples were fabricated using dipping times of 10 min and 40 min, and their morphology was investigated using AFM imaging.

For the nanocomposites made with GO, a 166 mg/L suspension of GO in DI water was prepared. The solution was ultrasonicated for an hour to ensure the dispersion of GO nanosheets. After depositing the cap layer as previously explained, the substrate was dipped in the positively-charged polymer solution with calcium acetate for 20 min, rinsed with DI water, dipped in the GO solution for 20 min, rinsed with DI water, dipped in the negatively-charged polymer solution with sodium silicate for 20 min and rinsed with DI water to form a trilayer. This procedure was repeated until the desired number of trilayers were assembled. Samples containing GO were fabricated using PEI/PSS polymers and were labelled PEI/PSS-CSH-GO.

### 2.3. AFM Imaging

The morphology of the samples was studied using a TT-AFM from AFMWorkshop (Signal Hill, CA, USA). A silicon probe from AppNano (Mountain View, CA, USA) with a cantilever length and width of 225 μm and 40 μm, respectively, a tip radius of less than 10 nm and a spring constant of 36–90 N/m was used for imaging. Images of each sample were taken in the non-contact mode.

### 2.4. X-ray Diffraction (XRD)

XRD was used to detect the CSH phase in the CSH/polymer nanocomposite. The XRD scans were conducted using a Siemens XRD with the Cu Kα radiation at a scan rate of 0.3 degrees/min and 0.02 degrees/step.

### 2.5. Fourier Transform Infrared Spectroscopy

The FTIR analysis was conducted on the samples at different stages of fabrication. A PerkinElmer Paragon 1000 FTIR (PerkinElmer, Boston, MA, USA) equipped with an ATR accessory was used at a scan rate of 4 cm^−1^ and in the range of 650 cm^−1^–4000 cm^−1^.

### 2.6. Zeta Potential Measurement

In this study, a Malvern Zetasizer was used to measure the charge and size of the polymers, polymer complexes and CSH/polymer particles in solutions. The concentration of polymers and salts in the solutions prepared for zeta potential measurement was the same as that of the solutions used to prepare the PEI/PSS and PDDA/PAA samples. Solutions containing CSH nanoparticles were also made by mixing equal amounts of positively-charged and negatively-charged polymer complex solutions. The pH of the solutions was determined using a pH meter.

### 2.7. AFM Nanoindentation 

AFM nanoindentation was performed on the samples with 25 bilayers or trilayers (in the case of samples containing GO) to determine their Young’s modulus. In an AFM indentation test, the AFM probe with known cantilever stiffness indents into the sample and then retracts from the surface. The deflection of the cantilever is logged and then converted to distance units using a sensitivity factor. Sensitivity is measured by performing the test on a sapphire substrate where the indentation is assumed to be negligible. The force-distance curves are obtained by converting the deflection data to force using Hooke’s law. The amount of deflection corresponding to the cantilever response is subtracted from the total deflection to generate the force-indentation distance. The unloading portion of the force-indentation curve was fitted using the Hertz model [47] as follows:

(1) $$F=\frac{4}{3}MR^{0.5}\delta^{1.5}$$

(2) $$\frac{1}{M}=\frac{1-\nu_{1}^{2}}{E_{1}}+\frac{1-\nu_{2}^{2}}{E_{2}}$$

(3) $$\frac{1}{M}=\frac{1-\nu_{1}^{2}}{E_{1}}$$

where F is the force in nN, δ is the indentation distance (nm), R is the tip radius in nm, M is the indentation modulus, $\nu_{1}$ and $E_{1}$ are the Poisson’s ratio and Young’s modulus (GPa) of the material and $\nu_{2}$ and $E_{2}$ are the Poisson’s ratio and Young’s modulus (GPa) of the AFM tip. Because the Young’s modulus of the diamond probe is much larger than that of the material being tested, Equation (2) is further reduced to Equation (3). A cube corner diamond probe (DNISP from Bruker (Camarillo, CA, USA)) with a spring constant of 291 N/m and a tip radius of 40 nm was used for AFM indentation. Indentation tests were performed on about 30 points with 5-µm spacing in 3 random frames for each sample.

## 3. Results and Discussion

### 3.1. Characterization

**Figure 3:**
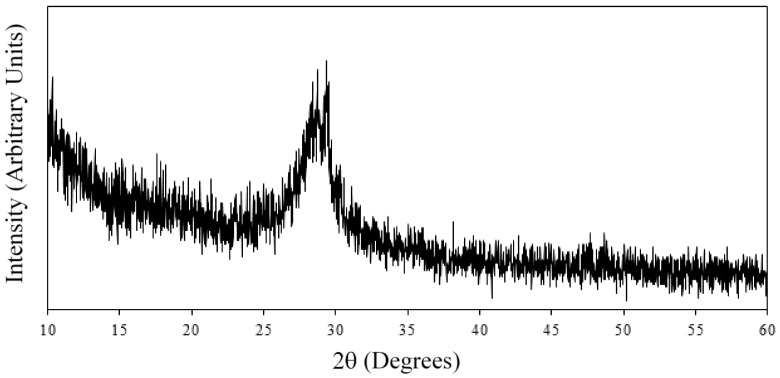
XRD spectrum of the PEI/PSS-CSH sample with three bilayers deposited on a silicon wafer substrate.

According to Guo et al. [26], during the LBL process, calcium and silicate ions in polymer solutions react and form CSH. The CSH particles formed in each bilayer affect the morphology of the surface [25,26]. The formation of CSH particles during the LBL assembly was explored using XRD as shown in Figure 3. Three bilayers of the PEI/PSS-CSH sample were deposited on a silicon wafer substrate prepared in the same way as explained for the glass slide substrate. The dipping time for each layer was 20 min. The silicon wafer was used because of its smoother surface compared to the glass slide so that the amount of noise was reduced significantly. and the thin layer of CSH was detected by XRD. The peak corresponding to CSH can be observed at 29.1° [48] indicating the formation of CSH in the nanocomposite. It should be noted that, according to available literature, the XRD spectrum of CSH exhibits other peaks with the strongest peak typically occurring at 29.1° [49]. Due to a very thin layer of CSH/polymer, other peaks are not evident in the XRD spectrum shown in Figure 3.

**Figure 4:**
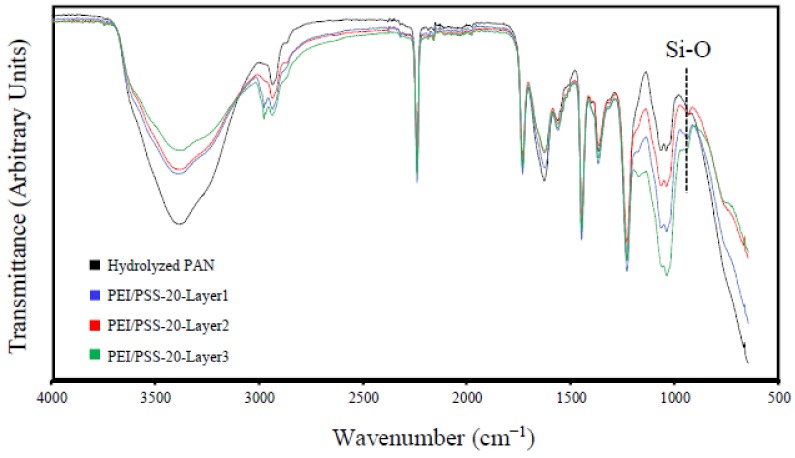
FTIR spectra of the hydrolyzed PAN and the PEI/PSS-CSH samples with 1, 2 and 3 bilayers.

The FTIR scans for the hydrolyzed PAN substrate and the PEI/PSS-CSH sample after deposition of each bilayer are shown in Figure 4. The O-H band at 3390 cm^−1^ and C=O band at 1632 cm^−1^ in hydrolyzed PAN decreased in intensity as more layers were deposited on the sample. The C-H band of polymers at 2980 cm^−1^ and the band at 1178 cm^−1^ corresponding to the sulfonate moiety of PSS [25] appeared after depositing the first bilayer and increased in intensity as the number of layers increased. The peak at 968 cm^−1^ is detectable after the deposition of the second bilayer and increased in intensity by deposition of the third bilayer. This peak corresponds to the Si-O stretching band in CSH [50] and indicates the increased formation of CSH particles as the number of bilayers increased. The peak at 1071 cm^−1^ in hydrolyzed PAN is attributed to the bending vibration of C-N group [51]. It is observed that the broad peak in the 1000–1200 cm^−1^ region, which is the characteristic absorption band of Si-O-Si asymmetric stretching [52], increased by increasing the number of layers, which provides evidence for the formation of CSH in the sample.

The results of the pH, zeta potential, and particle size measurements are provided in Table 1. It is seen that the solution of PEI-Ca^2+^ and PSS-SiO_3_^2−^ has a zeta potential of 10 mV and −42.9 mV, indicating the positive and negative charge of the polymer solution after the addition of salts, respectively. By mixing the PEI-Ca^2+^ and PSS-SO_3_^2−^ solutions, the size of the particles was increased to 205 nm, which indicates the formation of CSH nanoparticles in the aqueous solution. The charge of PEI/PSS-CSH solution is seen to be negative and slightly lower than that of PSS- SO_3_^2−^ due to the strong anionic character of PSS. The size of particles in PEI/PSS-CSH is also larger than that of PEI/PSS without Ca^2+^ or SO_3_^2−^, which provides evidence for agglomeration of CSH particles in this solution. The negative charge of the particles in PEI/PSS-CSH was measured to be higher than that of PEI/PSS; this indicates the negative charge of the silicate chains in CSH formed in situ during LBL deposition. 

The device was not able to measure the size of particles after mixing the PDDA-Ca^2+^ and PAA-SO_3_^2−^ solutions. This might be because of the formation of large agglomerated CSH particles. It is noted that the agglomerated particles were clearly observed in the mixed solution. The charge of particles in PDDA/PAA-CSH is also negative.

### 3.2. Morphology Examination

**Figure 5:**
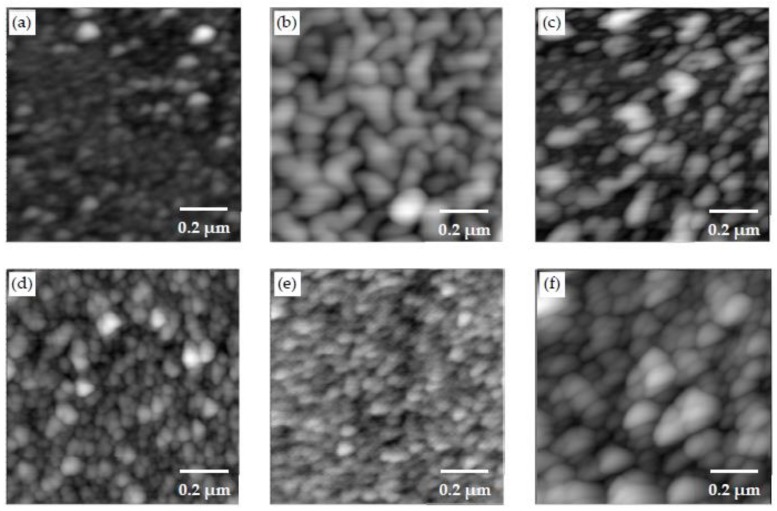
AFM scans of the (**a**) PEI/PSS; (**b**) PDDA/PAA; (**c**) PEI/PAA; (**d**) PEI/PSS-CSH; (**e**) PDDA/PAA-CSH and (**f**) PEI/PAA-CSH samples made of three bilayers.

The AFM images of the PEI/PSS, PDDA/PAA and PEI/PAA samples are shown in Figure 5a–c, respectively. Figure 5d–f shows the AFM scans of the PEI/PSS-CSH, PDDA/PAA-CSH and PEI/PAA-CSH samples. The LBL assembly of different sets of polymers revealed different types of morphology. However, when Ca^2+^ and SiO_3_^2−^ ions were added to the different polymer sets, globular features were detected in all the samples. The size and roughness of these particles were different in the samples. The roughness values reported in this paper are arithmetic roughness (R_a_), calculated as the arithmetic average of the surface height deviations measured from the mean plane. The roughness values of the samples over areas of 5 μm by 5 μm and 1 μm by 1 μm are shown in Table 2. Particles in the PDDA/PAA-CSH sample were denser compared to the other samples, and the roughness value was also the smallest among the samples. This was not expected in light of the particle size analysis of the mixture of the PDDA-Ca^2+^ and PAA-SO_3_^2−^ solutions, as shown in Table 1, where large agglomerates were observed. This difference could be attributed to the layer-by-layer deposition of the solutions. Particles in the PEI/PAA-CSH sample appeared larger than the other samples, which is also reflected in the increased roughness value of this sample. These observations indicate the important role of different charged polymers on the formation, growth and assembly of CSH particles. One explanation for this observation can be related to the conformation of polymer molecules in the layered structure of the samples. In PDDA/PAA-CSH, the polymer molecules may adopt a flat conformation and provide a template for growth and assembly of CSH particles. This can be attributed to the strong cationic PDDA, which maintains high positive charge density even at the pH of the solution, which is measured to be 10–11. On the other hand, PEI is a weak cationic polymer and is partially charged at the pH of the solutions, and it adopts a coil like conformation. Thus, it is expected that the growth and assembly of CSH in the coil-like template result in a larger CSH aggregate and increased porosity and less packing density of the nanocomposites, as demonstrated by increased roughness in the PEI/PSS-CSH and PEI/PAA-CSH samples. It is seen that the roughness of PEI/PSS and PEI/PAA is lower than that of PEI/PSS-CSH and PEI/PAA-CSH, respectively. It is interesting to note an opposite trend in the case of PDDA/PAA, where there is a reduction in roughness in PDDA/PAA-CSH. PAA is a weak electrolyte and is partially ionized below a pH of 6.5 [53]. Thus, at pH = 2.9, which corresponds to the solution of PDDA/PAA, its molecular structure consists of a segmental population of loops and tails [53]. Thus, this promotes the formation of large island-like features on the surface of the PDDA/PAA sample, which also leads to elevated roughness, as seen in Table 2.

**Figure 6:**
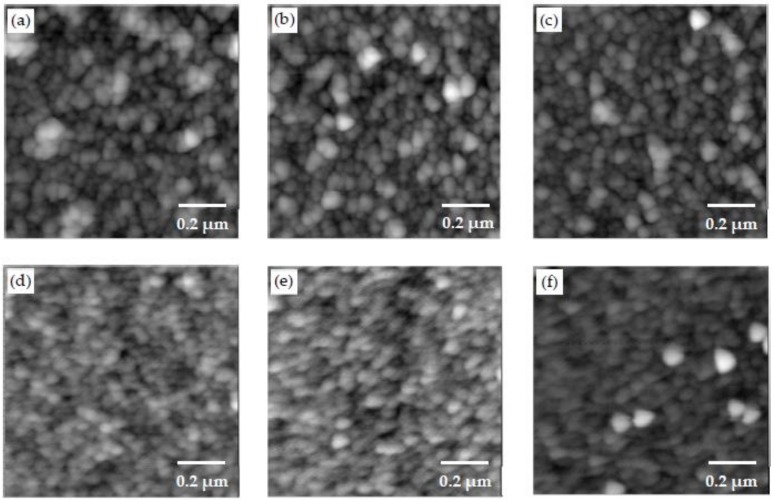
AFM images showing the morphology of (**a**–**c**) PEI/PSS-CSH(10), PEI/PSS-CSH and PEI/PSS-CSH(40), respectively. AFM images showing the morphology of (**d**–**f**) PDDA/PAA-CSH(10), PDDA/PAA-CSH and PDDA/PAA-CSH(40), respectively.

**Figure 7:**
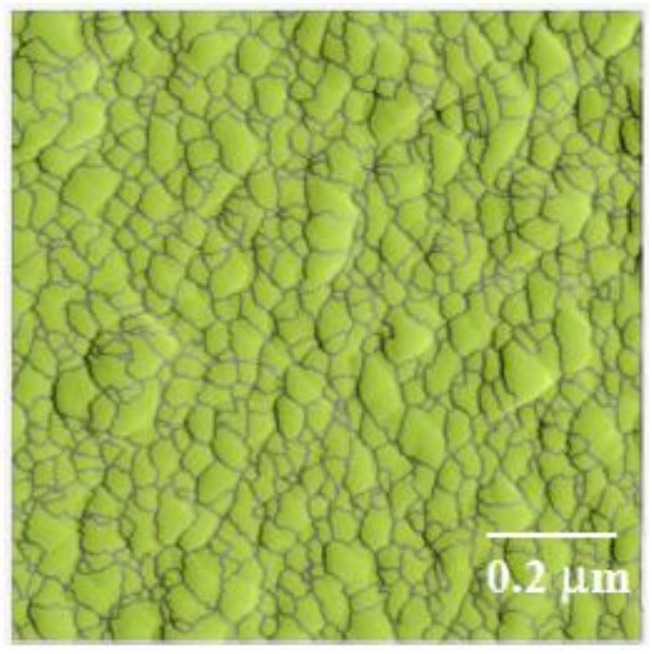
Segmentation of the AFM scan of PEI/PSS-CSH(10) using the watershed algorithm.

The AFM images of the samples prepared by different dipping times of 10 min, 20 min and 40 min are shown in Figure 6a–c. The number in parentheses in the sample designation corresponds to the duration of dipping during the fabrication of the samples. When not included in the sample designation, it implies a dipping time of 20 min, which was used for all other samples. Gywddion [54], an open source software to analyze the scanning probe microscopy (SPM) data, was used to apply the watershed algorithm to AFM scans in order to identify the particles and segment the images. The segmented image for one of the AFM scans is shown in Figure 7. The number of particles, as well as their mean area were obtained using this method. The roughness values of the samples and the number and mean area of particles in the 1 μm by 1 μm scans are shown in Table 3. By increasing the dipping time, the number of particles slightly increased, and the roughness value of the samples decreased. The increase in dipping time allowed more particles to form, and the denser morphology of the layers decreased the roughness value. A height profile (not shown here) clearly indicated that the height of the particles in the PDDA/PAA-CSH samples is much smaller than that of the PEI/PSS-CSH samples. This also explains the smaller roughness value of the PDDA/PAA-CSH samples compared to that of the PEI/PSS-CSH samples.

**Figure 8:**
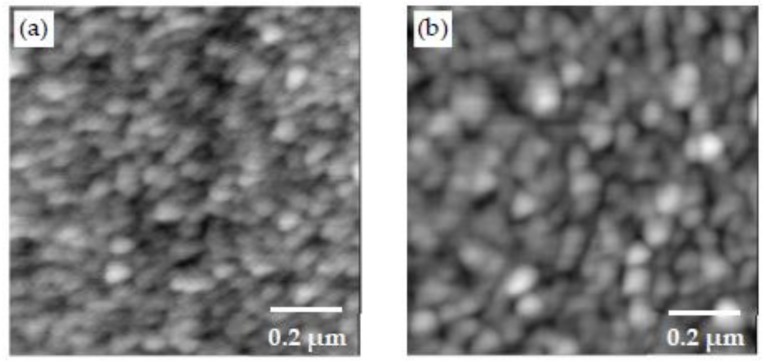
AFM image of (**a**) PDDA/PAA-CSH and (**b**) PDDA/PAA-CSH-HighpH made of three bilayers.

In order to examine the effect of pH, the PDDA/PAA-CSH-HighpH sample was prepared with the pH of the solutions raised to 12.5 by the addition of a 5 M NaOH solution. PDDA has a high positive charge density, which was reported to be unaffected by pH variation [55]. Its resistance to pH makes PDDA a good candidate for preparing the CSH/polymer nanocomposites at high pH. The AFM images of PDDA/PAA-CSH and PDDA/PAA-CSH-HighpH are shown in Figure 8. The roughness values of the samples and the number and mean area of particles in the 1 μm by 1 μm scan are shown in Table 4. It is seen that the PDDA/PAA-CSH sample at high pH exhibits a higher roughness and noticeably larger particles compared to PDDA/PAA-CSH. The effect of pH increase on CSH precipitation from an aqueous solution was studied in the past and shown to influence the structure and composition of CSH [56]. Prior work has also indicated the dependence of CSH nanoparticle aggregation on the pH of the solution [57,58]. The increase in pH influences the nucleation of CSH and also increases the negative charge density of CSH, which affects the interaction forces between the CSH nanoparticles and also between the CSH nanoparticles and polymer molecules. These processes are expected to be responsible for increased roughness and almost two fold larger area of the particles in PDDA/PAA-CSH-HighpH at high pH compared to PDDA/PAA-CSH. It should be noted that PAA is fully ionized above pH of 6.5 [53] and is not expected to undergo conformational changes when pH increases from about 10–12.5, as is the case in our study. In addition, as mentioned previously, PDDA is a strong polyelectrolyte, and its charge density does not change with pH. Thus, it is unlikely that the conformational change of the polyelectrolyte contributes to the observed change in the roughness and particle area in the sample with increased pH.

**Figure 9:**
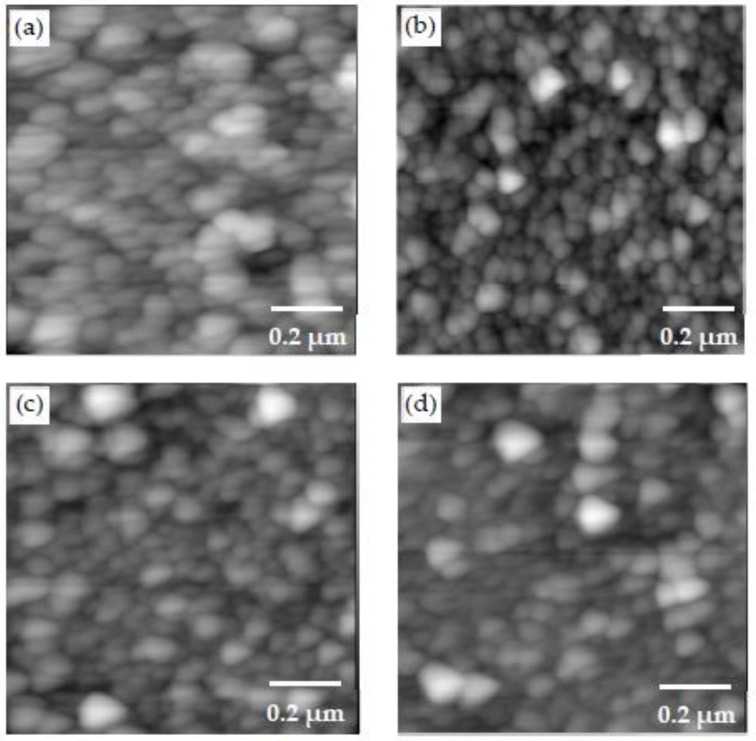
AFM images of (**a**) PEI/PSS-CSH-0.7; (**b**) PEI/PSS-CSH; (**c**) PEI/PSS-CSH-1.5 and (**d**) PEI/PSS-CSH-2.3 made of three bilayers.

The PEI/PSS-CSH samples at different C/S ratios were also studied to understand the effect of composition on the characteristics of the nanocomposites. It is well known that the C/S ratio is a determining factor in the properties of CSH [6]. These samples were prepared varying the concentration of the sodium silicate solution to obtain C/S ratios of 0.7, 1.5 and 2.3; these samples are labelled PEI/PSS-CSH-0.7, PEI/PSS-CSH-1.5 and PEI/PSS-CSH-2.3, respectively. The AFM images of the samples with different C/S ratios are shown in Figure 9. The image of PEI/PSS-CSH corresponding to the default C/S ratio of one is also included. The roughness values, the number, and mean area of particles in a 1 μm by 1 μm scan are shown in Table 5. No significant differences in the morphology of the samples can be observed. One possible explanation is that the change in the concentration of the silicate ions in the PSS-silicate solution does not lead to the formation of more PSS-silicate complexes in the range of silicate concentrations used in the experiment. In this case, the formed CSH particles on the surface would have a similar chemical structure and C/S ratios regardless of the concentration of sodium silicate in the solution. The other possibility is that the samples have different C/S ratios, but the morphology of the CSH particles is not dependent on the C/S ratio in the nanocomposite studied here. It should be mentioned that morphology dependence on C/S ratio can exist in nanocomposites with other polymers, and this needs further investigations. The effect of C/S on the atomic structure and mechanical properties of synthetic pure CSH powder has been studied in the past, and C/S has been shown to influence these characteristics of CSH [6]. However, direct morphology studies of synthetic CSH with varied C/S ratios are currently scarce. Transmission electron microscopy images of CSH powder with two different C/S were investigated in [59] however, no comparison in regard to the difference in morphology was made between the CSH samples with different C/S ratios studied in their paper.

It should be noted that the growth mechanism, linear or non-linear, of the LBL nanocomposites is important as it determines the final properties. The growth mechanism was not examined in this paper and will be investigated in future publications.

### 3.3. AFM Nanoindentation

**Figure 10:**
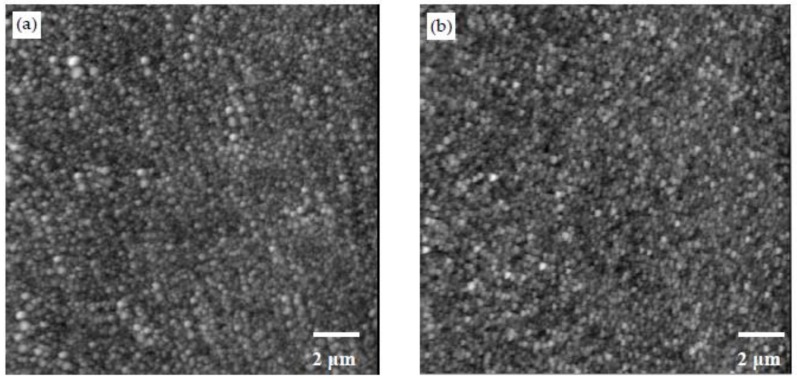
AFM images of PEI/PAA-CSH made of 25 bilayers (**a**) before and (**b**) after heat treatment.

**Figure 11:**
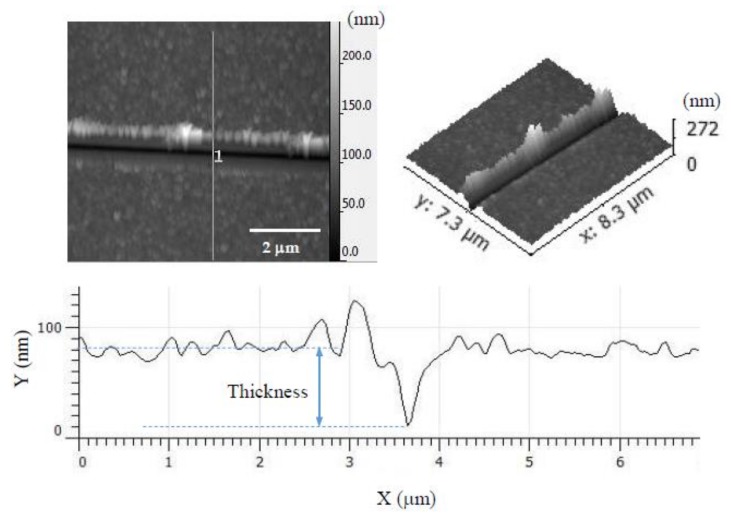
AFM images and height profile of the scratch made on the surface of PDDA/PAA-CSH with 25 bilayers after heat treatment.

To perform nanoindentation, the PEI/PSS-CSH, PDDA/PAA-CSH and PEI/PAA-CSH samples were made by the deposition of 25 bilayers to provide a sufficient thickness for indentation. To investigate the fraction of CSH in the nanocomposites, a heat treatment at 350 °C for 3 h was performed on the samples. The polymer portion of the samples was burnt out during the heat treatment, and CSH particles were left on the substrate. It should be noted that heat treatment at this temperature can affect the properties of CSH; however, such an effect was not considered in this study. The morphology of the PEI/PAA-CSH sample before and after heat treatment is shown in Figure 10. The roughness value of the sample before and after heat treatment was 9 nm and 8.5 nm, respectively, indicating an insignificant change in roughness as a result of heat treatment. The thickness of the deposited layers was measured before and after heat treatment. A scratch was made on the surface of the sample using the AFM probe. Then, the area around the scratch was scanned by AFM, and the thickness of the sample was measured. The AFM scan and a height profile of the scratch made on the PDDA/PAA-CSH sample after the heat treatment are shown in Figure 11. The results of the nanoindentation and thickness measurement for the samples made with different polymer complex sets are shown in Table 6. The thickness has decreased compared to the initial thickness in both samples after performing the heat treatment. The Young’s modulus of nanocomposites PEI/PSS-CSH and PDDA/PAA-CSH is similar and does not show a noticeable difference. The Young’s modulus of the samples before and after heat treatment is also listed in Table 6. It is seen that the Young’s modulus of the PEI/PSS-CSH sample increased modestly after heat treatment. The increase in the Young’s modulus after removal of polymers can be explained from the composite mixing rule [60] as the phase fraction of hard CSH is increased. However, no significant change was observed in the Young’s modulus value of the PDDA/PAA-CSH sample after performing the heat treatment. The nanoindentation of PEI/PAA-CSH also showed a small increase in the Young’s modulus after heat treatment. However, it should be noted that the roughness of PEI/PAA-CSH was large, so that reliable nanoindentation measurements of this sample were not able to be obtained, and for that reason, these values are not included in Table 6. 

The value of Young’s modulus of CSH/polymer nanocomposites after removal of polymer can be compared to those of CSH powder reported in the literature [11,15,61] (see Table 7). It should be noted that it is possible for the polymers to be incorporated in the atomic structure of CSH stacks in the form of surface adsorption or intercalation in the interlayer space [7]. It has been shown in the literature that the removal of the polymer interacting with CSH happens at a temperature range of 250 °C–550 °C [15]. Thus, the polymers incorporated in the atomic structure of CSH are not likely to be completely removed at the temperature used in the heat treatment. This is reflected by the lower values of the Young’s modulus of CSH after polymer removal compared to CSH powder as reported in the literature [11,15].

### 3.4. CSH/Polymer/GO Nanocomposite

As mentioned previously, LBL allows fabrication of nanocomposites with a nanoscale control over the fabrication process [22,23,24,25,26,27]. Thus, thin GO nanosheets are an ideal nanomaterial to study the effect of nanomaterials on CSH/polymer nanocomposites fabricated using LBL. To obtain insights into the effects of GO on CSH/polymer systems, the PEI/PSS-CSH-GO sample containing one trilayer of PEI-calcium acetate, GO and PSS-sodium silicate was prepared. The concentration of GO used in the fabrication of this sample was very low to prevent agglomeration of GO nanosheets in the nanocomposite. This way, a single GO nanosheet was able to be identified in the AFM images and allowed us to focus on the differences between the surface of GO and the surrounding regions.

**Figure 12:**
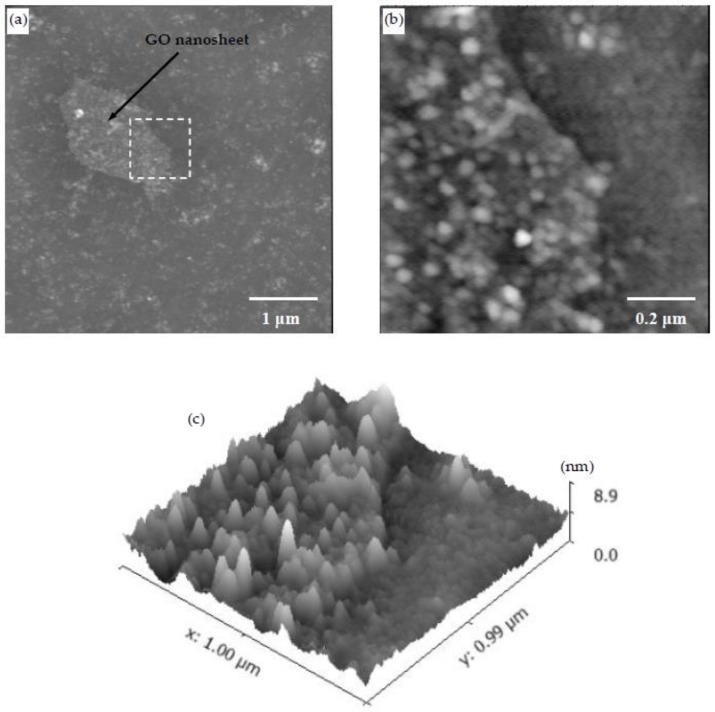
(**a**) AFM image showing a GO nanosheet in the PEI/PSS-CSH-GO sample made of one trilayer; (**b**,**c**) 2D and 3D higher magnification images of the area marked by a square in (**a**), respectively. A higher density of CSH particles is observed on the surface of GO.

The surface of the sample was scanned by AFM. The AFM scans of a GO nanosheet in the nanocomposite at different magnifications are shown in Figure 12a–c. It is observed that the concentration of the CSH particles on the surface of the GO nanosheet appears to be higher than the surrounding areas. This can be attributed to increased nucleation sites for the formation of CSH on the surface of GO. This observation can provide evidence at the nanoscale for increased hydration in cementitious materials as a result of the addition of GO [44]. Care should be taken in the interpretation of this observation as the effect of polymers cannot be decoupled in the LBL-fabricated CSH/polymer nanocomposite. An ideal situation would be to examine the nucleation of GO in LBL-fabricated CSH/GO systems without the presence of polymers, which seems impractical as the polymers are needed for the LBL deposition method.

**Figure 13:**
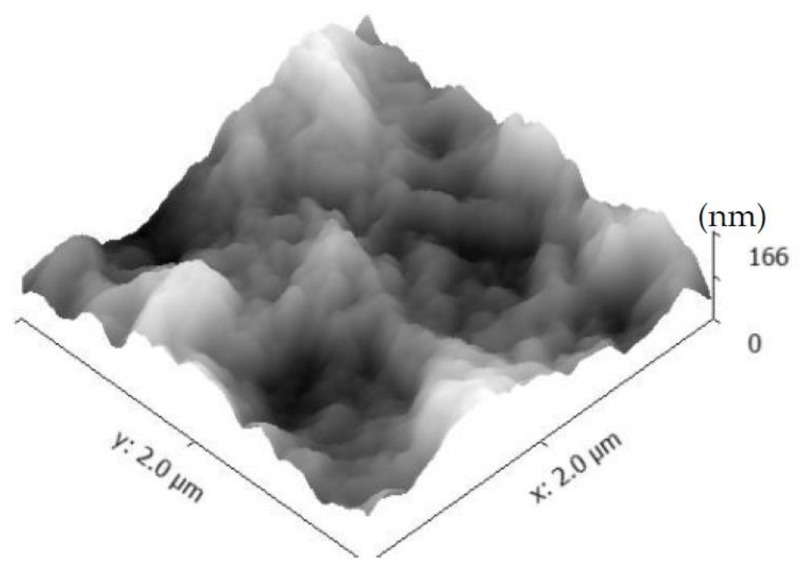
AFM image of PEI/PSS-CSH-GO showing the wavy morphology with increased roughness compared to that of PEI/PSS-CSH without GO nanosheets, as shown in Table 3.

The morphology of the PEI/PSS-CSH-GO samples made of 25 trilayers of GO, PEI-calcium acetate and PSS-sodium silicate is shown in Figure 13. The concentration of GO was also higher compared to that in the sample with only one trilayer to investigate the influence of GO on the overall properties of the nanocomposite. The surface of this sample appears to be rougher and irregular compared to that of the samples without GO. The surface roughness of the sample with GO is measured to be one order of magnitude higher than that of the sample without GO. The increase in roughness could be because not all GO nanosheets are deposited in a completely planar position, and they are folded or take a wavy form within the layers, as seen in Figure 13. The CSH particles can be observed in this figure. The folding of GO nanosheets results in a reduction in the packing density of the nanocomposite. It is worth mentioning that although GO nanosheets seem to increase the nucleation of CSH particles, the adopted morphology of GO, such as folding and waviness, could result in a loosely-packed microstructure of the nanocomposites, contributing to a lower Young’s modulus. More investigations are necessary to obtain more detailed insights into the CSH/polymer/GO systems.

## 4. Conclusions

CSH/polymer nanocomposites were synthesized with the LBL method, and their morphology and mechanical properties were investigated using AFM imaging and AFM nanoindentation. To elucidate the effect of GO nanosheets on CSH/polymer nanocomposites, GO nanosheets were incorporated in the LBL assembly of CSH/polymer nanocomposites. The findings of this study are as follows:

- Different morphologies were observed in samples made with different polymers, and this indicated the potential influence of polymers on the microstructure of CSH/polymer nanocomposites.
- An increase in the pH level was shown to result in samples with higher roughness and larger CSH particles.
- The change in C/S ratio in the range examined in this study did not appear to show a noticeable effect on the morphology of CSH/polymer fabricated by the LBL method. Further investigations are necessary to provide more insights into the effect of C/S.
- The Young’s modulus of CSH/polymer nanocomposites obtained from AFM nanoindentation was measured to be in the range of the Young’s modulus values of powder CSH/polymer reported in the literature [8,11]. In spite of observed differences in the morphology of PEI/PSS-CSH and PDDA/PAA-CSH, the nanoscale Young’s modulus of these nanocomposites did not exhibit significant differences.
- It was shown that GO nanosheets seemed to increase the nucleation of CSH particles as inferred from the higher density of CSH particles on a GO nanosheet compared to regions outside of the GO nanosheet.

## Figures and Tables

**Table 1:** pH, zeta potential value, and particle size of the polymer/ion solutions.

Solution	pH	Zeta Potential (mV)	Size (nm)
PEI-Ca^2+^	10.5	10.0	85.7
PSS-SO_3_^2−^	11.7	−42.9	63.4
PEI/PSS	10.5	−28.3	60.9
PEI/PSS-CSH	10.8	−39.3	205.0
PDDA-Ca^2+^	8.7	44.0	482.7
PAA-SO_3_^2−^	11.5	−53.5	20.1
PDDA/PAA	2.9	70.4	323.9
PDDA/PAA-CSH	10.1	−15.4	

**Table 2:** Roughness value of the samples made with only polymers and polymer complexes.

Sample Label	Ra (nm) 5 μm by 5 μm	Ra (nm) 1 μm by 1 μm
PEI/PSS	1.47	1.18
PEI/PSS-CSH	4.77	4.1
PDDA/PAA	7.95	6.85
PDDA/PAA-CSH	0.85	0.873
PEI/PAA	8.12	9.75
PEI/PAA-CSH	23.2	19.7

**Table 3:** Roughness value, number of particles, and mean area of the nanocomposites fabricated with different dipping times.

Sample Label	R_a_ (nm) 5 μm by 5 μm	R_a_ (nm) 1 μm by 1 μm	Number of Particles 1 μm by 1 μm	Mean Area of the Particles (nm^2^)
PEI/PSS-CSH(10)	7.5	6.13	720	1000
PEI/PSS-CSH	4.77	4.1	770	980
PEI/PSS-CSH(40)	4.42	3.82	791	950
PDDA/PAA-CSH(10)	1.03	1	640	1190
PDDA/PAA-CSH	0.85	0.873	678	1110
PDDA/PAA-CSH(40)	0.91	0.99	709	1060

**Table 4:** Roughness value, number of particles, and mean area of the CSH particles in the PDDA/PAA-CSH and PDDA/PAA-CSH-HighpH samples made of 3 bilayers.

Sample Label	R_a_ (nm) 5 μm by 5 μm	R_a_ (nm) 1 μm by 1 μm	Number of Particles 1 μm by 1 μm	Mean Area of the Particles (nm^2^)
PDDA/PAA-CSH	0.85	0.873	678	1110
PDDA/PAA-CSH-HighpH	1.46	1.39	394	2060

**Table 5:** Roughness value, number of particles, and mean area of the CSH particles in the PEI/PSS samples with different calcium to silicate (C/S) ratios made of three bilayers.

Sample Label	R_a_ (nm) 5 μm by 5 μm	R_a_ (nm) 1 μm by 1 μm	Number of Particles 1 μm by 1 μm	Mean Area of the Particles (nm^2^)
PEI/PSS-CSH-0.7	4.42	4.33	800	980
PEI/PSS-CSH	4.77	4.1	770	980
PEI/PSS-CSH-1.5	4.35	4.08	717	1060
PEI/PSS-CSH-2.3	4.00	4.03	711	1070

**Table 6:** Average Young’s modulus value and thickness of the samples comprising 25 bilayers made with different polymer complex sets.

Sample Label	E (GPa) Before Heat Treatment	Thickness (nm)	E (GPa) After Heat Treatment	Thickness (nm)
PEI/PSS-CSH	10.9	200	14.3	80
PDDA/PAA-CSH	11.9	180	11.0	80

**Table 7:** Young’s modulus of C-S-H obtained from previous studies.

References	E (GPa)
[11,15]	26.8
[61]	13–18.4
